# Annual Report U. S. M. Hosp. Service for 1875

**Published:** 1877-07

**Authors:** 


*Annual Report of the Supervising Surgeon General of the Marine Hospital Service of the United States for the fiscal year* 1875. By John M. Woodworth, M. D.

The usual very carefully prepared records and statistics are given with several contributed articles upon yellow fever, consumption, scurvy, the seton in paralysis and epelipsy, also one very well prepared upon syphilis and chanceroid.

